# Extracellular ATP, as an energy and phosphorylating molecule, induces different types of drug resistances in cancer cells through ATP internalization and intracellular ATP level increase

**DOI:** 10.18632/oncotarget.21231

**Published:** 2017-09-23

**Authors:** Xuan Wang, Yunsheng Li, Yanrong Qian, Yanyang Cao, Pratik Shriwas, Haiyun Zhang, Xiaozhuo Chen

**Affiliations:** ^1^ Department of Biological Sciences, Ohio University, Athens, Ohio 45701, USA; ^2^ Interdisciplinary Graduate Program in Molecular and Cellular Biology, Ohio University, Athens, Ohio 45701, USA; ^3^ The Edison Biotechnology Institute, Ohio University, Athens, Ohio 45701, USA; ^4^ Department of Chemistry and Biochemistry, Ohio University, Athens, Ohio 45701, USA; ^5^ Department of Biomedical Sciences, Heritage College of Osteopathic Medicine, Ohio University, Athens, Ohio 45701, USA

**Keywords:** the Warburg effect, tumor microenvironment, cancer metabolism, PDGFR, sunitinib

## Abstract

Cancer cells are able to uptake extracellular ATP (eATP) via macropinocytosis to elevate intracellular ATP (iATP) levels, enhancing their survival in drug treatment. However, the involved drug resistance mechanisms are unknown. Here we investigated the roles of eATP as either an energy or a phosphorylating molecule in general drug resistance mediated by ATP internalization and iATP elevation. We report that eATP increased iATP levels and promoted drug resistance to various tyrosine kinase inhibitors (TKIs) and chemo-drugs in human cancer cell lines of five cancer types. In A549 lung cancer cells, the resistance was downregulated by macropinocytosis inhibition or siRNA knockdown of PAK1, an essential macropinocytosis enzyme. The elevated iATP upregulated the efflux activity of ABC transporters in A549 and SK-Hep-1 cells as well as phosphorylation of PDGFRα and proteins in the PDGFR-mediated Akt-mTOR and Raf-MEK signaling pathways in A549 cells. Similar phosphorylation upregulations were found in A549 tumors. These results demonstrate that eATP induces different types of drug resistance by eATP internalization and iATP elevation, implicating the ATP-rich tumor microenvironment in cancer drug resistance, expanding our understanding of the roles of eATP in the Warburg effect and offering new anticancer drug resistance targets.

## INTRODUCTION

Drug resistance is a major problem in cancer therapies and responsible for most relapses, one of the major causes of death in cancer [1]. Several cancer drug resistance mechanisms have been identified, and more than one drug resistance mechanism may be present in a single tumor [2]. Among these mechanisms, multi-drug resistance (MDR) is known to be ATP-dependent [3, 4], as ATP is used as an energy source for the ATP-Binding Cassette (ABC) transporters to pump drugs out of cancer cells [5, 6]. ATP is also known to be involved in other types of drug resistance. Cancer cells have been shown to have higher intracellular ATP (iATP) levels than their non-cancerous counterparts *in vitro* [7, 8]. Furthermore, drug-resistant cancer cell lines exhibit even higher iATP levels than the non-resistant cancer cell lines from which the resistant cell lines are derived [9, 10]. These findings strongly suggest that higher iATP levels are closely associated with cancer cells and appear to be a necessary condition for the phenotype and drug resistance state of cancer cells. However, it was not known that extracellular ATP (eATP) contributes to drug resistance in cancer until we recently reported, for the first time, that eATP substantially elevated iATP concentration and significantly enhanced the survival of non-small cell lung cancer (NSCLC) A549 cells treated by tyrosine kinase inhibitors (TKIs) [8]. More significantly, increased survival was observed when eATP concentrations used were in the range of the reported intratumoral extracellular ATP concentrations *in vivo* [8, 11–14], demonstrating potential clinical relevance of the phenomenon. We further showed that the iATP level elevation is largely mediated by three endocytic processes: macropinocytosis, clathrin- and caveolae-mediated endocytoses, macropinocytosis being predominant [15]. Uptake of nutrients in the tumor microenvironment by macropinocytosis and other mechanisms has recently been named as an emerging hallmark of cancer metabolism [16]. Consistent with this characterization, an ATP-sharing model was proposed to explain roles of eATP in eATP-induced increase in cancer cell growth rate and survival [17]. However, which drug resistance mechanisms that are induced by eATP is not known. It is also unclear if the eATP-induced drug resistance is a general phenomenon present in cell lines of different cancer types as well as *in vivo*. Based on our preliminary observations and the fact that ATP functions in cells as both an essential energy source and a signal-generating molecule by phosphorylating proteins involved in signal transduction, we hypothesized that eATP induces multiple types of drug resistance to both chemo and target drugs in various cell lines of different cancer types by eATP internalization and subsequent iATP level elevation.

To determine how eATP induces drug resistance, we examined changes in the viability and iATP levels of five cancer types treated with either TKIs or chemo drugs in the presence or absence of eATP. We also investigated changes in phosphorylation of proteins involved in platelet-derived growth factor receptor (PDGFR) signaling (a major signal transduction pathway in A549), as well as how eATP affects the MDR activity of cancer cell lines. Additionally, we studied changes in apoptosis, and rates of glycolytic and mitochondrial oxidative phosphorylation (OXPHOS) of A549 cells incubated with or without eATP in the presence or absence of sunitinib to determine the effects and mechanism of eATP-induced drug resistance to sunitinib. Sunitinib, in this study, was used as a representative TKI that binds to the ATP binding site of the target receptor tyrosine kinases (RTK) and inhibits RTK by preventing ATP from binding to the same site, resulting in reduction of RTK phosphorylation [18, 19]. Sunitinib was injected in the presence or absence of ATP into xenografted A549 tumors to study eATP-induced protein phosphorylation *in vivo*. Chemical inhibitors and siRNA knockdown were used to determine the involvement of macropinocytosis in eATP internalization and drug resistance. The results of this study have identified two previously uncharacterized eATP-induced drug resistance mechanisms that have significant biological and clinical ramifications in drug resistance and in developing new strategies fighting against drug resistance in cancer.

## RESULTS

### Extracellular ATP induced drug resistance and iATP level elevation in multiple cell lines of several cancer types to both target and chemo drugs

The first study was to determine if the eATP-induced drug resistance and iATP level changes are present in multiple cancer cell lines of various cancer types, and reduce effectiveness of both target and chemo drugs. As shown in Tables 1 and 2, 1mM eATP induced drug resistance and iATP elevation in most of the five cell lines of five cancer types tested. ABC transporters expressed by different cell lines and responsible for the efflux of different drugs are summarized in Supplementary Tables 2 and 3. By comparing the iATP level and drug resistance status, a correlation was found between the elevated iATP level and drug resistance for a given cell line. This correlation was found in 4 out of 5 cancer cell lines and cancer types with an exception of HT-29 colon cells, in which iATP level was not elevated by eATP.

**Table 1:** Extracellular ATP promotes resistance to multiple drugs in various cell lines

	Sunitinib	Sorafenib	Gefitinib	Erlotinib	Imatinib	Paclitaxel	Cisplatin	Doxorubicin
A549 (lung)	++	-	+	+	-	+	-	+
SK-HEP-1 (liver)	++	++	++	-	+	+	+	++
MCF7 (breast)	-	+	++	+	+	+	+	+
HT-29 (colon)	+	-	+	-	+	-	-	-
PANC-1 (pancreas)	-	+	+	-	+	+	+	+

Note: Cancer cell lines of five origins were treated with different drugs with or without 1mM ATP for 24h. Then cell viability was measured by MTT assay. Cancer cell viability increased by ATP was calculated and designated as -: <5%, +: 5-20%, and ++: >20%. The increases with “+” were all significant.

**Table 2:** Intracellular ATP levels of five cancer cell lines treated with or without eATP

	Sunitinib	Sorafenib	Gefitinib	Erlotinib	Imatinib	Paclitaxel	Cisplatin	Doxorubicin
A549 (lung)	++	-	+	+	++	+	+	+
SK-HEP-1 (liver)	++	++	++	++	++	++	++	++
MCF7 (breast)	-	++	++	+	++	+	+	+
HT-29 (colon)	-	-	-	-	-	-	-	-
PANC-1 (pancreas)	++	++	-	+	-	+	+	+

Note: Cancer cell lines of five origins were treated with different drugs with or without 1mM ATP for 24h. Then intracellular ATP level was measured by ATP assay. Intracellular ATP level increased by ATP was calculated and designated as -: <10%, +: 10-20%, and ++: >20%. The increases with “+” were all significant.

### Extracellular ATP increased survival and decreased apoptosis of A549 cells treated with sunitinib

NSCLC A549 cells were selected as a model cell line for studying eATP-induced drug resistance mechanisms due to their frequent use in cancer metabolism and ATP research [20], and demonstrated drug resistance [8, 15, 21]. A549 cells also exhibit PDGFR-expressing and macropinocytosis phenotypes [8, 22]. In our previous study, we observed that eATP substantially elevated intracellular ATP levels of A549 cells and rescued them from the inhibition of sunitinib [8], serving as the basis for using sunitinib as a representative TKI in the mechanism study.

**Figure 1:**
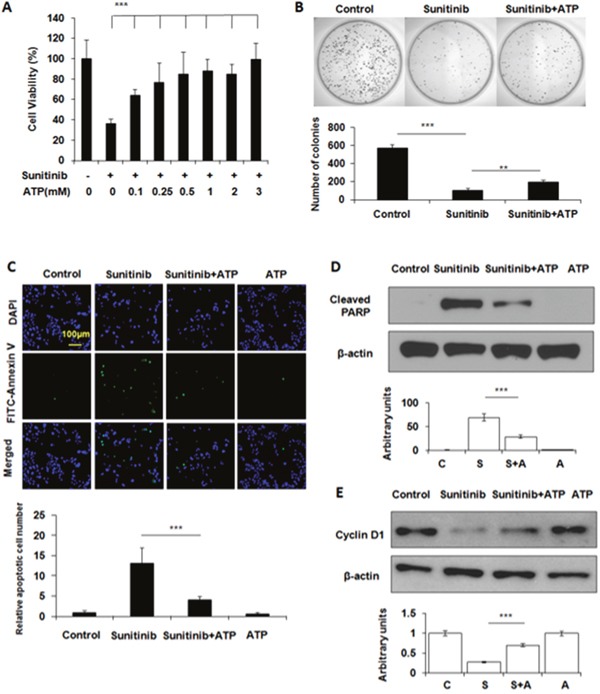
Extracellular ATP rescues A549 cells treated with sunitinib A549 cells were treated with 20 μM sunitinib in the presence or absence of ATP at various concentrations for various times. After the treatment, cells were measured for their viability with MTT assay, the clone-formation capability with the clonogenic assay, and cell survival by apoptosis assay and Western blots. Data is reported as mean ± standard deviation. ^**^ = p < 0.01, ^***^ = p < 0.001. **(A)** ATP dose-dependent cell viability of A549 cells treated with sunitinib. **(B)** Determination of the relative differential survival rates of A549 cells treated by sunitinib in the presence or absence of 1mM ATP by the clonogenic assay. **(C)** Extracellular ATP reduced sunitinib-induced apoptosis as shown by the immunochemical staining of A549 cells treated with sunitinib in the presence or absence of 1 mM ATP. Green-fluorescent cells are apoptotic cells. **(D)** Extracellular ATP reduced PARP cleavage as shown by Western blot. **(E)** Extracellular ATP partially restored levels of cell cycle protein cyclin D1 suppressed by sunitinib as shown by Western blot.

From 0.1 to 0.5 mM, eATP increased the survival rates of A549 cells treated with 20 μM sunitinib in a dose-dependent manner in a 24-hour MTT assay (Figure 1A). The survival rate did not further increase at eATP concentrations higher than 0.5 mM. This was qualitatively consistent with our previous observation that eATP enhanced drug resistance to sunitinib and pazopanib, two representative TKIs [8]. The difference between this and earlier studies was that this study extended the tested range of ATP concentrations by including concentrations either below or in the range of intratumoral extracellular ATP concentration reported previously [11–14]. The increased survival was also confirmed by a clonogenic assay, in which significantly more survived cells from sunitinib/eATP-treatment eventually formed more clones compared to sunitinib-treatment alone (Figure 1B).

Sunitinib kills cancer cells by inducing apoptosis. Therefore, numbers of apoptotic cancer cells with or without eATP were determined by apoptosis staining. Results showed that sunitinib-treated A549 cells displayed increased cell apoptosis compared to untreated cells while eATP treatment substantially reduced the sunitinib-induced apoptosis (Figure 1C). The reduction of apoptosis was also confirmed by the PARP protein cleavage assay, which revealed that the sunitinib treatment resulted in PARP cleavage while eATP significantly reduced the cleavage (Figure 1D). ATP also partially restored the level of cyclin D1 suppressed by sunitinib (Figure 1E).

### Extracellular ATP induced intracellular ATP level elevation regardless of the presence of sunitinib

**Figure 2:**
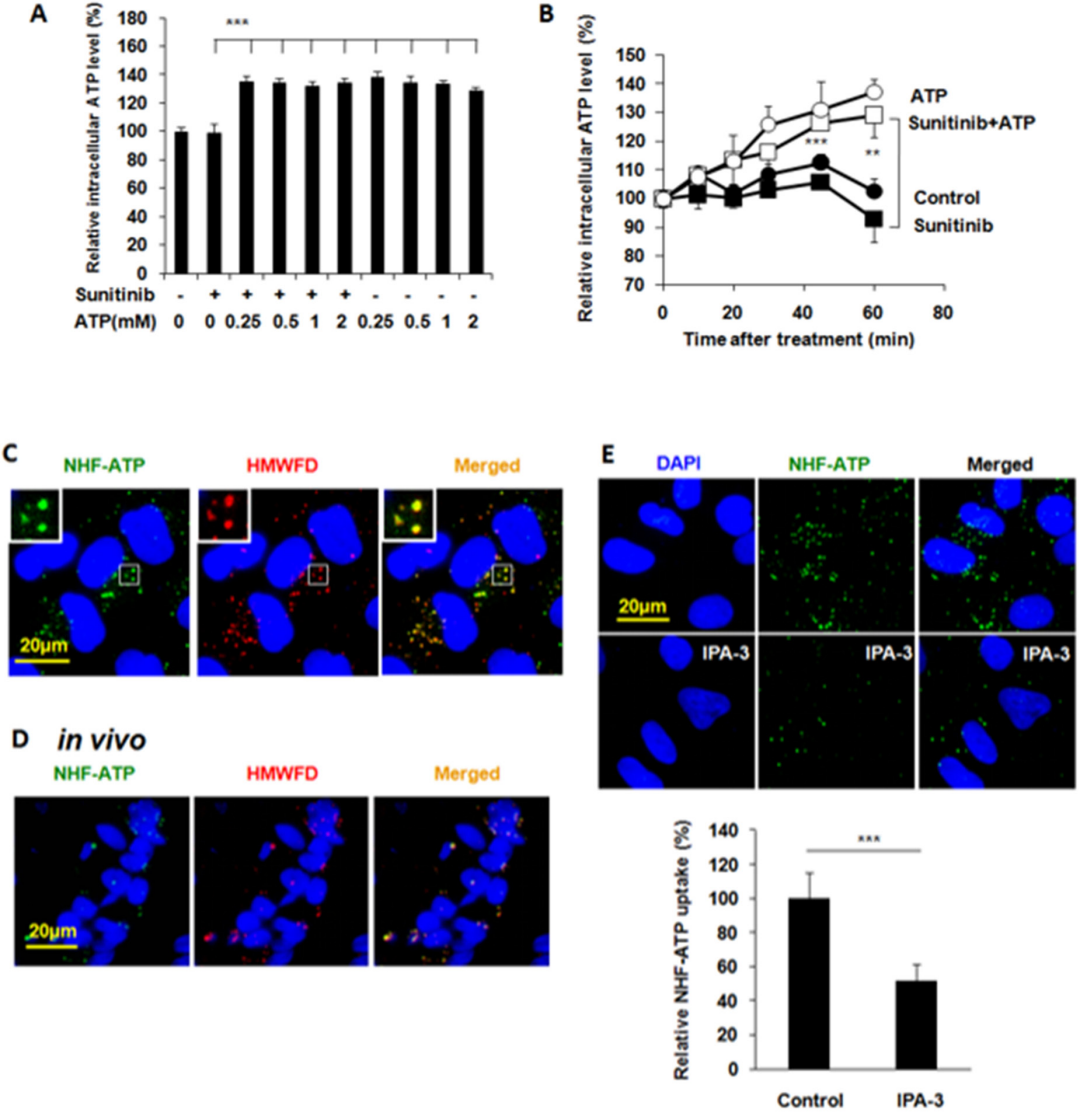
Extracellular ATP elevates intracellular ATP levels and A549 cells internalize NHF-ATP both *in vitro* and *in vivo* primarily using macropinocytosis A549 cells were treated with 20 μM sunitinib in the presence or absence of ATP at various concentrations for various times. After the treatment, cells were measured for intracellular ATP levels with an ATP assay. For ATP internalization studies, A549 cells on coverslips or tumors grown on nude mice were treated / injected with NHF-ATP (green) in the presence or absence of high molecular weight fluorescent dextran (HMWFD, red) for various times. After the treatment and fixation, cells or tumors were visualized with fluorescent microscopy and analyzed by Image J. Data is reported as mean ± standard deviation. ^**^ = p < 0.01, ^***^ = p < 0.001. **(A)** Extracellular ATP induced intracellular ATP level elevation in A549 cells treated with or without sunitinib for one hour. **(B)** Extracellular ATP (1mM) induced intracellular ATP level elevations in A549 cells in a time-dependent manner with or without 20 μM sunitinib. **(C)** A549 cells internalize NHF-ATP and HMWFD through macropinocytosis *in vitro*. Merged photo and inlet show the colocalization of NHF-ATP and HMWFD (yellowish spots). NHF-ATP internalization was also observed in H1299 cells (Supplementary Figure 1). **(D)** A549 tumors co-internalize NHF-ATP and HMWFD *in vivo*. **(E)** A549 cells internalize significantly less NHF-ATP when treated with a macropinocytosis inhibitor IPA-3 (50 μM).

Next, we wanted to find out how eATP treatment led to the reduced apoptosis and increased survival. The ATP assay revealed that these changes were accompanied with eATP-induced iATP level elevations (Figures 2A and 2B). Interestingly, 0.25 mM eATP induced as much iATP increase as 0.5 or 1 mM eATP one hour after the start of the incubation (Figure 2A). In a time course study, the iATP levels were found to increase in a time-dependent manner and as early as 10 min after the addition of eATP (Figure 2B). Based on all these results, we speculated that the observed increase in cancer cell survival or drug resistance was due to the elevated iATP levels induced by eATP and the elevated iATP levels were generated by eATP internalization.

### A549 cells and tumors internalized NHF-ATP

**Figure 3:**
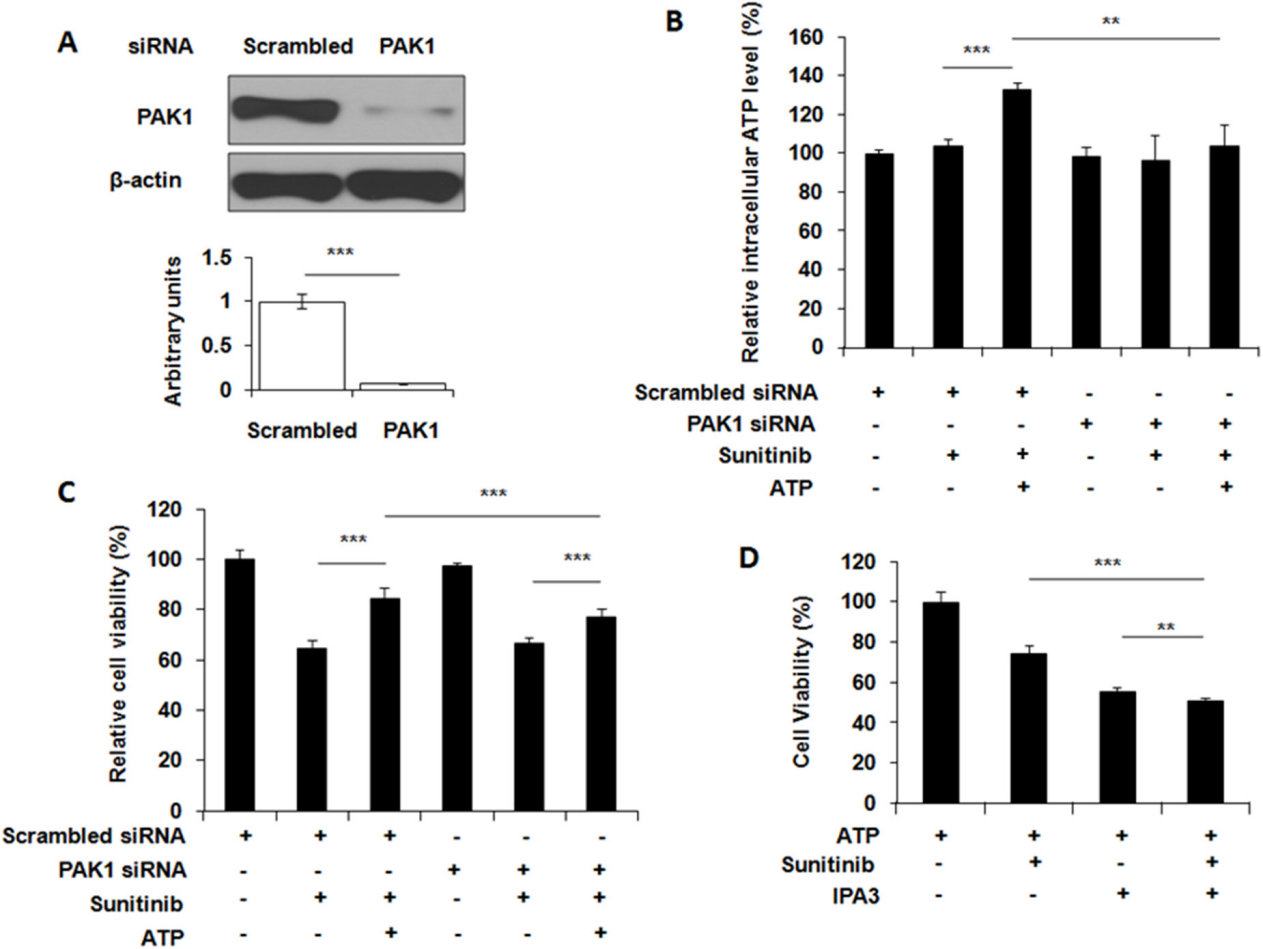
Blocking macropinocytosis reduces extracellular ATP-induced iATP increase and cell survival A549 cells were either transfected with siRNA targeting PAK1 or treated with macropinocytosis inhibitor IPA3. After transfection or inhibitor treatment, cells were assayed for the PAK1 levels by Western blots, or treated with 20 μM sunitinib in the presence or absence of 1 mM ATP followed by either cell viability assay or ATP assay. Scrambled siRNA was used as a control. Data is reported as mean ± standard deviation. ^**^ = p < 0.01, ^***^ = p < 0.001. **(A)** Knockdown of PAK1 by a verified PAK1-specific siRNA with scrambled siRNA as PAK1 base line control and β-actin as protein loading control. **(B)** Relative intracellular ATP levels in PAK1 siRNA-knocked down A549 cells treated with or without 10 μM sunitinib in the presence or absence of 1 mM eATP. **(C)** Relative cell viability of PAK1 siRNA knocked down A549 cells treated with or without 10 μM sunitinib in the presence or absence of 1 mM eATP. **(D)** Relative cell viability of A549 cells treated by 10 μM sunitinib with or without 50 μM IPA3 in the presence or absence of 1 mM eATP.

ATP is very unstable and rapidly turned over in cells. Therefore, it is very difficult to monitor ATP movement directly. We previously showed that the nonhydrolyzable fluorescent ATP (NHF-ATP) can be used as a surrogate of ATP to study ATP movement across the cell membrane [15]. When NHF-ATP (green) was added together with HMWFD (red) to A549 cells, they colocalized in vesicles inside A549 cells as demonstrated by the yellowish color of the merged vesicle image (Figure 2C). A similar observation was made for lung cancer H1299 cells (Supplementary Figure 1). This indicates that NHF-ATP was internalized along with HMWFD by macropinocytosis as HMWFD is a macropinocytosis tracer [15, 23]. The ATP internalization was also shown in tumors, indicating this process occurred *in vivo* (Figure 2D). The NHF-ATP internalization was suppressed by the treatment of IPA3, a macropinocytosis inhibitor (Figure 2E), further confirming that the internalization was mediated by macropinocytosis. The involvement of macropinocytosis in the mechanism of ATP internalization was further supported by an siRNA knockdown of PAK1, an enzyme intimately involved in macropinocytosis [24]. The knockdown resulted in reduction of PAK1 protein levels (Figure 3A), iATP levels (Figure 3B), as well as survival of eATP- and sunitinib-treated A549 cells compared with no knockdown samples (Figure 3C). Consistent with the siRNA knockdown result, when macropinocytosis inhibitor IPA3 was used in sunitinib-treated A549 cells in the presence of eATP, IPA3 further reduced the viability of A549 cells (Figure 3D). Taken together, it was concluded that A549 cells’ intracellular ATP level was elevated by internalizing eATP primarily via macropinocytosis.

### Extracellular ATP restored phosphorylation level of proteins involved in the PDGFR signaling pathways in sunitinib-treated A549 cells

**Figure 4:**
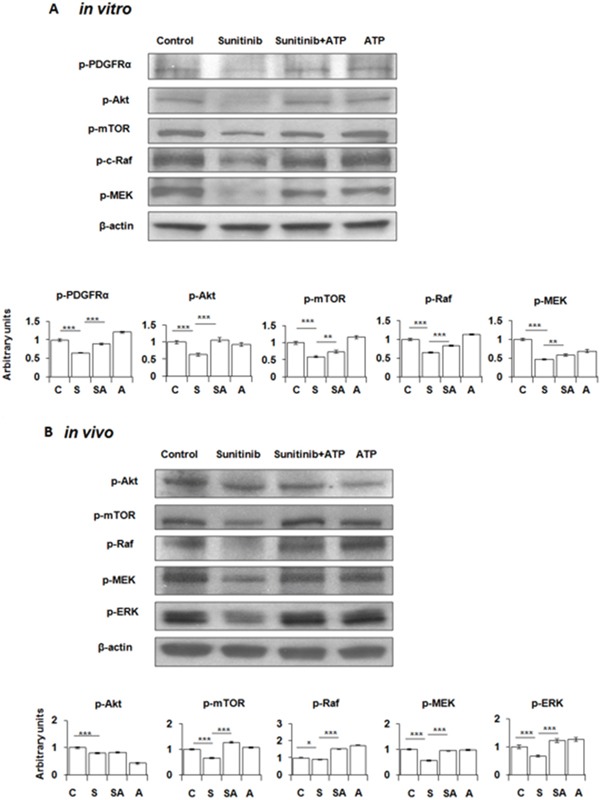
Extracellular ATP partially restores phosphorylation levels of PDGFR and proteins in the PDGFR-mediated signaling pathways suppressed by sunitinib A549 cells or tumors were treated or injected with or without 20 μM sunitinib in the presence or absence of 1 mM ATP for 30 min (*in vitro*) or 6 min (*in vivo*). After treatments, cells/tumors were lysed for isolation of total proteins, which were analyzed by Western blots. β-actin samples served as loading controls for protein normalization. Data is reported as mean ± standard deviation. ^*^ = p < 0.05, ^**^ = p < 0.01, ^***^ = p < 0.001. **(A)** Phosphorylation pattern of PDGFR and key proteins in the PDGFR- Akt-mTOR and the PDGFR-Raf-MEK pathways in A549 cells. **(B)** Protein phosphorylation pattern of the treated A549 tumors. Control = C, Sunitinib = S, ATP = A.

Next, we determined the mechanism of enhanced cell survival mediated by the eATP-induced intracellular ATP level elevation. Because sunitinib inhibits PDGFR [18, 19] and A549 cells highly express PDGFR as the only one of several known RTK targets of sunitinib [22], we speculated that the increased iATP level could increase phosphorylation of PDGFR and proteins downstream of PDGFR-mediated signaling pathways. Western blot analysis revealed that, as expected, sunitinib reduced phosphorylation of PDGFRα and other key proteins involved in the two PDFGR-mediated cell growth / survival signaling pathways: PDGFR-Akt-mTOR pathway and PDGFR-Raf-MEK pathway (Figure 4A). In contrast, eATP at 1 mM partially restored the sunitinib-suppressed phosphorylation of PDGFR and downstream proteins. These results suggest that extracellular ATP increased survival of sunitinib-treated A549 cells by partially restoring reduction of phosphorylation levels of proteins involved in cell growth / survival signaling pathways.

### Intratumoral extracellular ATP also reversed sunitinib-suppressed phosphorylation of proteins involved in cell growth/survival signaling pathways in A549 tumors

The *in vivo* study with ATP-injected A549 tumors removed from nude mice revealed that the phosphorylation patterns of proteins involved in the PDGFR-mediated signaling pathways in tumors were very similar to those found in cultured A549 cells: sunitinib treatment resulted in reduced protein phosphorylation levels and eATP partially restored them (Figure 4B). These results strongly suggest that the eATP-induced resistance to sunitinib also occur *in vivo*.

### Extracellular ATP promoted efflux activity of ABC transporters

**Figure 5:**
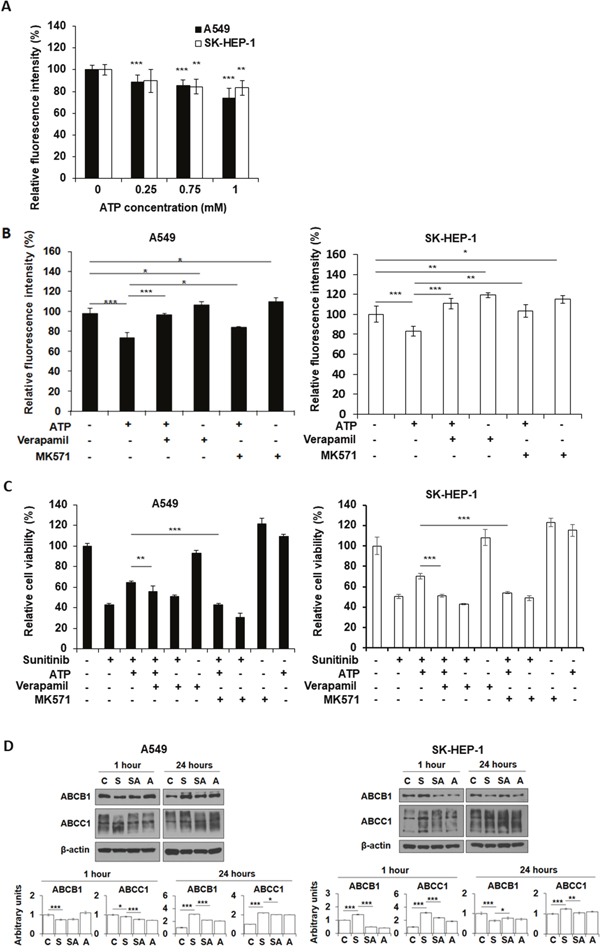
Extracellular ATP upregulates activity of ABC transporters A549 or SK-HEP-1 cells were treated with either ATP of various concentrations and/or specific inhibitors of ABC transporters in the presence or absence of sunitinib. After treatment, cells were measured for either retained fluorescence dye or cell viability by MTT assay. Data is reported as mean ± standard deviation. ^*^ = p < 0.05, ^**^ = p < 0.01, ^***^ = p < 0.001. **(A)** Extracellular ATP reduced florescence intensity of the dye (increased the efflux activity of ABCB1 and/or ABCC1). **(B)** Specific ABC inhibitors increased the retention of fluorescence dye. **(C)** Inhibitors of ABCB1 and ABCC1 reduced the viability of A549 and SK-HEP-1 cells treated by 20 μM sunitinib in the presence or absence of 1 mM extracellular ATP. Verapamil: 20 μM; MK-571: 50 μM. **(D)** Western blot analyses of protein levels of ABCB1 and ABCC1 in A549 or SK-HEP-1 cells treated by sunitinib in the presence or absence of 1 mM ATP. Untreated cells serve as baseline controls.

ATP is not only a signal-generating molecule, but also an energy molecule. We speculated that eATP-induced iATP increase may also upregulate MDR activity in cancer cells. The fluorometric MDR assay, which measures dye retained inside cells, revealed that eATP enhanced ABC transporters’ efflux activity in A549 and SK-HEP-1 cells by pumping intracellular dye out of cells compared to the no ATP control (Figure 5A). The reduction in intracellular dye concentration was approximately 10% for 0.25 mM eATP and approximately 20% for 1 mM eATP, showing a dose-dependent trend. This assay specifically measures ABCB1 and ABCC1 mediated activity. Thus, the assay results indicate that eATP enhanced efflux activity of ABCB1 and ABCC1, which are known to be expressed in A549 [25, 26] and SK-HEP-1 cells [27, 28]. The second MDR assay with specific MDR inhibitors showed that verapamil and MK-571 increased intracellular dye concentrations (Figure 5B), confirming the involvement of the ABCB1 and ABCC1 in the eATP-induced efflux activity in two cancer cell lines. The MTT assay confirmed the fluorometric MDR assay results by showing that eATP enhanced viability of A549 cells treated by sunitinib (Figure 5C, bar 3 vs. bar 2 from left) and the enhancement was reduced by addition of MDR inhibitors verapamil and MK-571 (bar 4 vs. bar 3, and bar 7 vs. bar 3). These results suggest that ABCB1 and/or ABCC1 efflux activity was upregulated by eATP in A549 and SK-HEP-1 cells, decreasing intracellular sunitinib accumulation and augmenting resistance to the drug. Involvement of ABCG2 was also determined with its inhibitor novobiocin, and the results indicate that ABCG2 did not have detectable contribution to the drug resistance in A549 cells (data not shown).

It is conceivable that eATP, as an energy and signaling molecule, not only upregulates iATP levels and enhances ABC transporters’ efflux activities, but may also regulate ABC transporter activity at the gene expression level. To test this, protein levels of the ABC transporters involved were measured. Western blot analyses revealed that, one hr after treatment, A549 cells showed reduction in levels in both ABCB1 and ABCC1 in sunitinib treated cells, while ATP treatment slightly increased ABCB1 but significantly lowered ABCC1 (Figure 5D). In contrast, at 24 hours after the treatment, sunitinib treated A549 cells increased levels of both ABCB1 and ABCC1 by 2-3 fold, while ATP treatment reduced the increase. Importantly, ATP also significantly reduced the ABCC1 levels in sunitinib treated cells compared to cells treated by sunitinib alone in both cell lines (Figure 5D). It was concluded that eATP treatment significantly altered protein levels for the ABC transporters involved in drug efflux.

### Purinergic receptor signaling was not involved in ATP-induced drug resistance

**Figure 6:**
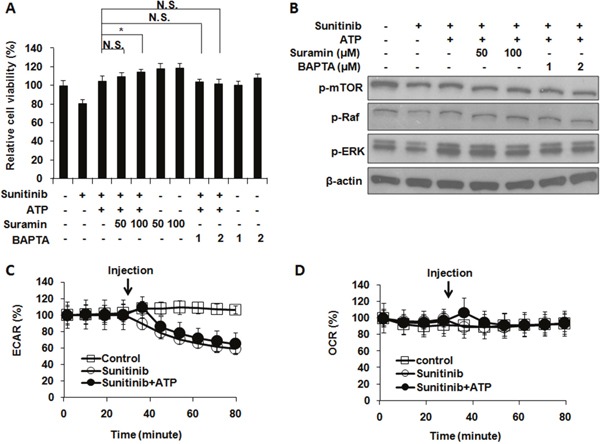
Extracellular ATP-induced drug resistance does not involve general purinergic receptor signaling or ECAR and OCR rates A549 cells were treated with 10 μM sunitinib in the presence or absence of purinergic receptor inhibitors with or without 1 mM extracellular ATP. After treatment, cells were measured for their viability and the phosphorylation pattern of proteins involved in PDGFR signaling pathway. Alternatively, sunitinib-treated cells with or without ATP were monitored by the Seahorse analyzer for their extracellular acidification rates (ECAR) or oxygen consumption rates (OCR). Data is reported as mean ± standard deviation. ^*^ p < 0.05. **(A)** Purinergic receptor (PR) signaling did not significantly alter viability of A549 cells treated with sunitinib and/or ATP. **(B)** PR signaling did not significantly change phosphorylation pattern of proteins involved in PDGFR-mediated signaling pathways. **(C)** ECAR were not changed when A549 cells were treated with or without ATP except for one time point (the point of immediately after the ATP injection (as indicated by arrow) and the change lasted no more than 15 min). **(D)** OCR were not changed except for the time point of immediately after the ATP injection.

Purinergic receptor (PR)-mediated signaling has been known to influence intracellular metabolism [29, 30], so general inhibitors of PR were used to determine if PR signaling is involved in the resistance. Cell viability (MTT) assays reveal that treatments of PR signaling inhibitors suramin or BApTA did not reduce cell viability (Figure 6A), indicating that overall PR signaling does not positively contribute to cell growth or survival in this process. Therefore, PR signaling as a whole does not contribute to the eATP-related drug resistance in A549 cells. This was also confirmed by a western blot analysis on proteins from A549 cells treated with sunitinib with or without eATP in the presence or absence of PR inhibitors (Figure 6B). These results were consistent with our previous findings that PR signaling as a whole does not significantly contribute to eATP internalization [15].

### Intracellular ATP synthesis did not contribute to ATP-mediated drug resistance

Metabolic rate analysis was used to determine if intracellular ATP synthesis contributed to the iATP level increase. The results demonstrated that, after addition of 1 mM eATP, small and temporary increases of glycolysis (ECAR) rates were observed, which lasted no more than 15 min (Figure 6C). Similar changes were observed for the mitochondrial OXPHOS (OCR) rates (Figure 6D). These small and very short-term rate changes are insufficient to account for the eATP-induced iATP level elevation and enhanced cell survival. Therefore, the elevated iATP levels, and the concomitant drug resistance, were not the result of increased glycolytic or mitochondrial ATP synthesis in A549 cells.

## DISCUSSION

Naming opportunistic uptake of nutrients by cancer cells from tumor microenvironment through macropinocytosis and other mechanisms as one of the hallmarks of cancer metabolism [16] indicates the prevalence of and essential role played by this phenomenon in cancer metabolism. Recent studies have revealed that cancer cells within tumors are genetically and metabolically heterogeneous [31–33]. Cancer cells can be normoxic or hypoxic depending upon their locations relative to blood vessels in the tumor [31–33]. As a result, the ways and amount of ATP produced by a cancer cell in a tumor vary tremendously depending upon its oxygen status and other nutrition supplies [34]. To compete for and exploit the microenvironment resources, cancer cells are known to accelerate the ATP synthesis rate (ATP synthesized per unit of time) by sacrificing ATP production yield (ATP synthesized per unit of substrate) through drastically upregulating glycolysis [34, 35]. It is known that intracellular ATP levels of cancer cells are higher than those in normal cells of the same tissue origin [7, 8] and drug-resistant cancer cells have even higher ATP levels than drug-sensitive cancer cells from which the resistant cells were derived [9, 10]. Thus, higher iATP levels are closely associated with cancer cells and even with drug resistant phenotype. In addition, cancer and stromal cells work together to create a tumor microenvironment that is substantially different from those in normal tissues and essential to tumor development [36, 37]. Ample experimental evidence indicates that greater-than-previously-expected exchanges of signals and materials are present among cancer cells and between stromal and cancer cells [38–40]. Concentrations of intratumoral extracellular ATP (eATP) have been measured in the range of very high μM to low mM, being ~ 10^3^ times higher than those found in the normal tissues of the same origin [11–14] and similar in concentrations to circulatory amino acids, proteins, and glucose [39, 41], is one of the important intratumoral extracellular molecules potentially being exchanged and plays previously unrecognized important functions in tumorigenesis and other processes such as drug resistance. An “ATP sharing model” was proposed to account for this phenomenon [15, 17]. The recent discovery of ATP’s novel hydrotropic activity in normal cells [42] also implies similar activity of iATP with even higher concentrations in cancer cells.

As shown by Table 1, eATP induced drug resistance in all five cell lines in five different cancer types tested. In these cell lines, eATP induced drug resistance to several target drugs as well as chemo drugs. These results show that eATP-induced drug resistance appears to be a general phenomenon among different types of cancer and anticancer drugs. On the other hand, the specific drug resistance mechanisms induced by eATP might be cell line-dependent, since each cell line may express different RTKs and ABC transporters. This is the first time the eATP-induced general drug resistance is described.

eATP also increased iATP levels in four of five cancer cell lines (Table 2). More significantly, increased iATP was found to correlate with drug resistance status when the ABC transporters expressed by the cell line matched those required for the efflux of a given drug. These results were consistent with our original hypothesis that the drug resistance was due to eATP-induced iATP level elevation. Some drugs do not correlate with the iATP level changes. These may be largely due to incompatibility between the ABC transporters that efflux these drugs and the ABC transporters expressed in those specific cell lines. Other unknown mechanisms may also be involved.

Various assays revealed that eATP increased A549 survival, reduced apoptosis and protein cleavage of apoptosis-inducing PARP, and upregulated Cyclin D1 expression (Figure 1). These results, taken together, suggest eATP is likely to reduce apoptosis of A549 cells induced by sunitinib through downregulating PARP cleavage and upregulating cyclin D1-mediated cell cycle progression, a pathway known to be mediated initially by PDGFR signaling for cell survival.

Several mechanisms may simultaneously be present in A549 cells for mediating eATP-induced drug resistance. (i) eATP may function exclusively extracellularly, without entering cells, by activating cell membrane associated receptors and then their downstream intracellular signaling pathways. (ii) eATP is somehow internalized and functions intracellularly, and (iii) both above-described mechanisms are present in the same cells. Elevation of iATP levels by a wide range of eATP concentrations regardless the presence of sunitinib (Figure 2A) and time-dependent and rapid iATP level elevations induced by eATP (Figure 2B) suggested the possibility of eATP induced the elevation by entering cells. The elevation was detected as early as 10 min after the addition of eATP. Fluorescence internalization assays in cultured A549 cells and tumors provide strong visual evidence that the second mechanism, eATP internalization, is present in both A549 cells and tumors. These results significantly expand the functional concentration range of eATP in cancer and exhibit the dynamic nature of the iATP level elevation mediated by eATP in the initial phase of the ATP internalization. This finding is also consistent with our conclusion drawn from our previous study that eATP is internalized by cancer cells through macropinocytosis and other endocytic processes [8, 15], as these processes internalize ATP regardless of eATP concentrations relative to iATP levels. Finally, the macropinocytosis-mediated ATP internalization and its contribution to A549 cells’ increased survival, i.e., drug resistance, is further supported by the siRNA knockdown of PAK1 (Figure 3A-3C) and IPA3 inhibitor study (Figure 3D). For these reasons, eATP should be considered as a key extracellular nutrient along with glucose, amino acids, lipids, proteins and others for cancer cells in tumors to take up, since intratumoral extracellular ATP is abundantly present in tumors [11–14]. It is noteworthy that eATP is the only molecule that can be taken up and directly used for energy and signal generation without additional biochemical reactions. It is “free lunch” for cancer.

The next question we tried to address was: exactly how the elevated iATP levels induce drug resistance. This question could be approached based on the nature of TKIs used in this study. Many TKIs work by inhibiting cell membrane-associated receptor tyrosine kinases (RTKs) in cancer. For example, sunitinib functions as an ATP competitor and inhibits PDGFR and/or other RTKs by binding to the ATP-binding site located on the intracellular domains of RTKs [18, 19]. Consequently, inactivated RTKs stop transmitting growth signals to their downstream protein factors, leading to cancer cell growth suppression or even apoptosis.

It is conceivable that significantly increased iATP levels could promote cell survival by increasing phosphorylation/activation of RTKs and downstream proteins involved in cell growth and survival in a stoichiometric manner. This hypothesis was supported by the observations that, in eATP-treated A549 cells, sunitinib suppressed phosphorylation of PDGFR and key proteins involved in the PDGFR-Akt-mTOR and PDGFR-Raf-MEK signaling pathways (Figure 4A). These two pathways are known to lead to cell growth, proliferation, and survival. In comparison, ATP partially but substantially restored the suppression. Also, the phosphorylation site on PDGFR is intracellularly located, implicating the action site of ATP. Furthermore, similar protein phosphorylation changes were observed in ATP-injected A549 tumors (Figure 4B). These results indicate that the cell responses to treatment of sunitinib and eATP observed in cultured A549 cells also occur *in vivo*, strongly suggesting that the same eATP-induced drug resistance mechanisms are also present in tumors. It is noteworthy that all the target drugs included in this study bind to ATP binding site of different RTKs and are therefore direct ATP competitors.

On the other hand, target drugs such as sunitinib have much higher binding affinities to the ATP binding site than ATP [43, 44] (while iATP levels are much higher than that of sunitinib), suggesting that the increase in protein phosphorylation may involve not only iATP level elevation but also a second mechanism that lowers intracellular sunitinib concentration.

Fluorescent dye studies revealed that eATP increased activity of ABC transporters in a dose-dependent manner (Figure 5A) and the specific ABC transporters involved, as designated by the assay kit, are ABCB1 and ABCC1, which are expressed in both A549 [25, 26] and SK-HEP-1 cells [27, 28] (Supplementary Table 2). These studies also show that blocking drug efflux activity with inhibitors against specific ABC transporters can reduce the enhanced drug efflux (Figure 5B) and viability (Figure 5C) induced by eATP. Since ABC transporters’ activity is driven by ATP hydrolysis, and the ATP binding site (ATP action site) of ABC transporters are located on the inside of the cell membrane, this result provides another piece of evidence supporting the notion that eATP upregulates MDR activity by eATP internalization and the resultant elevated iATP levels.

Extracellular ATP not only enhanced ABC transport efflux by providing extra energy (dye efflux assay was a one hour incubation assay and might not have gene expression involved), it also altered protein levels of the ABC transporters in A549 and SK-HEP-1 cells in a 24 hr assay (Figure 5D). Particularly, eATP significantly reduced ABCC1 levels in both cell lines treated by sunitinib, suggesting that eATP-induced iATP level elevation may enhance ABCC1 efflux rate and reduce the total number of ABCC1 transporters for drug efflux activity. These results reveal that eATP exerts profound effects on cancer cells, modulating ABC transporter activities at both enzymatic activity rate and enzyme expression levels to potentially efflux sunitinib and enhance drug resistance.

The MDR assays offer additional mechanistic insights to the results in Table 1. First, whether eATP induces drug resistance in a given cancer cell line to a specific drug depends on which ABC transporters efflux that drug and if the cancer cell lines express those specific ABC transporters (Supplementary Tables 2 and 3). For example, eATP induces large drug resistance to sunitinib in A549 cells because sunitinib is effluxed by ABCB1 and ABCC1 while A549 cells express high levels of these two transporters. In contrast, eATP does not induce drug resistance to cisplatin in A549 cells, because cisplatin is effluxed by ABCC2 and ABCC3 but A549 cells do not significantly express these two transporters. In SK-HEP-1 cells, eATP induces very high iATP elevations. At the same time, SK-HEP-1 cells express ABCB1, ABCC1 and ABCC2. Thus, eATP was able to induce drug resistance in SK-HEP-1 cells to both sunitinib and cisplatin. Some cell lines, such as HT-29, and drugs appear not to conform to these rules. These may be due to incomplete information on the expressed ABC transporters for the cell lines and/or additional drug resistance mechanisms present in those cell lines such as alterations of gene expression of ABC transporters after eATP treatment.

When added to sunitinib-treated A549 cells in the presence of eATP, macropinocytosis inhibitor IPA3 further reduced viability of the cells (Figure 3D), indicating the potential of combinational treatment of a macropinocytosis inhibitor and a TKI for the anticancer efficacy enhancement. This result is consistent with the siRNA knockdown study (Figure 3). All of these results suggest that extracellular ATP and ATP uptake may have the potential to become new anticancer targets for those cancers that heavily depend on macropinocytosis for nutrient/ATP supply. This proposal is further supported by a study in which tumor growth was reduced by the application of apyrase, a type of ATPase, in an animal glioblastoma model [45].

**Figure 7:**
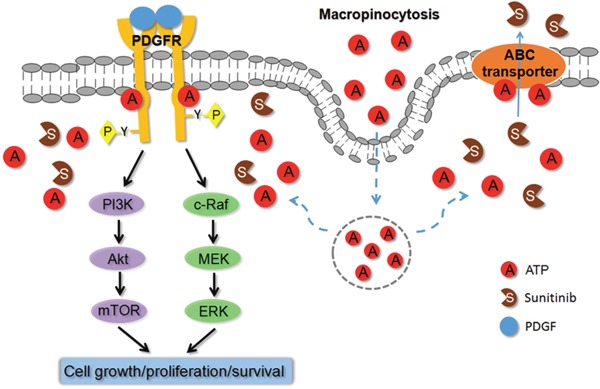
Hypothetical model for extracellular ATP-induced drug resistance to anticancer drugs Based on all experimental results described in this and previous studies on extracellular ATP, a model is proposed to explain the mechanisms of action by which extracellular ATP induces resistance to TKIs and chemo drugs. According to this model, extracellular ATP is internalized by cancer cells primarily via macropinocytosis, resulting in substantially elevated intracellular ATP levels. The elevated intracellular ATP molecules upregulate ABC transporter (drug-efflux) activities by providing more energy and altering protein levels and compete with the TKI molecules for the ATP binding site located on RTKs, leading to increased phosphorylation and activation of RTKs, such as PDGFR. The activated RTKs send more cell growth and anti-apoptosis signal down the signaling pathways, resulting in reduced cell death and increased cell survival. These do not exclude the possibility of additional mechanism(s) for eATP. More studies are needed to verify these mechanisms.

Based on all the results of our previous and current studies, a hypothetical model for the mechanism for the drug resistance induced by the extracellular ATP is proposed (Figure 7). According to this model, eATP is internalized by sunitinib-treated cancer cells through macropinocytosis to elevate iATP levels. The significantly elevated iATP will then upregulate ABC activity, by providing additional energy to the transporters or altering the gene expression levels of the transporters, pumping out sunitinib and lowering its intracellular concentration, and upregulating suppressed phosphorylation of RTK (PDGFR in this case) by sunitinib. The upregulation of phosphorylation of PDGFR in turn activates the two major PDGFR-mediated pathways involved in the increased drug resistance in A549 NSCLC cells: the Akt-mTOR and Raf-MEK pathways. The augmented phosphorylation/activation of PDGFR and the proteins in the pathways leads to reduced apoptosis, increased cell cycle progression and cell proliferation, and increased survival of the anticancer drug-treated cancer cells. This hypothetical model may also apply to other cancer cell lines and other TKIs or chemo drugs, but specific RTK and ABC involved are cell line-dependent. The identification of eATP-induced drug resistance significantly deepens and expands our understanding of ATP’s roles in cancer metabolism, especially the Warburg effect [8, 15, 17]. The recently described hydrotropic activity of ATP in normal cells [42] is also supportive to this model, since it implies that intracellular ATP may not be evenly distributed inside cells but is more concentrated at subcellular regions where protein concentrations are particularly high. It is conceivable that ATP concentrations around ABC transporters and TKRs in cancer cells may be higher than other intracellular locales so that ATP performs its energy and phosphorylating functions where it is also needed for maintaining protein solubility. More studies are needed before this model can be verified.

In summary, this study identifies a new player in the cancer drug resistance - intratumoral extracellular ATP. eATP-induced drug resistance appears to be very common in cancer cell lines of different cancer types and is likely to be found in other cancer types. In addition, two specific drug resistance mechanisms have been identified, elevated phosphorylation of proteins involved in cell growth and cell survival – RTK-mediated signaling pathway and augmented ABC (MDR) activity. Both mechanisms are driven by eATP-induced iATP level increase through macropinocytosis-facilitated eATP internalization. These two identified mechanisms are different from those previously described ATP-related drug resistance mechanisms [9, 10] in that these are intrinsic in nature while those earlier described mechanisms are acquired. Also, the origins of ATP for the resistance are different. Considering that intratumoral extracellular ATP levels are in the range of 0.5 to 1 mM and the intracellular ATP levels are significantly elevated when extracellular ATP is present, the contribution of ATP to these new types of drug resistance is substantial. Therefore, the inhibition of the ATP internalization and eATP degradation in principle is able to greatly reduce the drug resistance and enhance the anticancer efficacy of TKIs and chemo drugs.

## MATERIALS AND METHODS

### Compounds, cancer cell lines, and antibodies

Human NSCLC A549 cells, breast adenocarcinoma MCF7 cells, colorectal adenocarcinoma HT-29 cells, pancreas ductal adenocarcinoma PANC-1 cells, and hepatic adenocarcinoma SK-HEP-1 cells were purchased from ATCC and propagated according to ATCC specified conditions. Sunitinib, imatinib, sorafenib, gefitinib, erlotinib, paclitaxel, cisplatin, doxorubicin and ATP were purchased from Sigma. Nonhydrolyzable fluorescent ATP (NHF-ATP) was from Jena Bioscience (Germany). High molecular weight fluorescent dextran (HMWFD) was from Invitrogen. Antibodies against phosphorylated and total Akt, mTOR, ERK1/2, PAK1, cleaved PARP, cyclin D1 and β-actin were from Cell Signaling. Antibodies against phosphorylated and total PDGFRα, c-Raf and MEK were from Santa Cruz Biotechnology.

### ATP and cell viability/proliferation assays and Western blot analyses

The luciferase activity-based ATP assay was performed to measure iATP levels and the MTT assay was performed to determine viability and iATP levels of five human cancer cell lines of five cancer types treated with or without sunitinib or other chemo or target drugs in the presence or absence of ATP at various concentrations (0.1 - 1 mM) as previously described [8, 21]. The concentrations of different drugs used were based on reported IC_50_ values of the drug in different cancer cell lines (See Supplementary Table 1 for the used drug concentrations). The measured iATP levels of the untreated cell samples (negative controls) expressed in relative luminescence units (RLUs) or the absorbance of untreated cells in the MTT assay were assigned a relative value of 100%, and all other measured iATP levels or MTT absorbance values were normalized by and compared to their respective controls.

Western blot analyses were performed to measure changes in phosphorylation levels of key proteins involved in PDGFR-mediated signaling pathways or protein levels of ABC transporters in A549 and SK-HEP-1 cells treated with or without sunitinib in the presence or absence of 1 mM eATP. β-actin was used as loading control to normalize protein or phosphorylation signals.

### Fluorescence microscopy

ATP internalization studies were performed with NHF-ATP (green fluorescence) or a HMWFD (red fluorescence) or together as described previously [8, 15]. A549 or H1299 lung cancer cells grown on cover slips were serum-starved for 18 hrs, washed with PBS, and then incubated with serum-free DMEM containing 10 μM NHF-ATP or 8 mg/ML HMWFD in the presence or absence of IPA-3, a macropinocytosis inhibitor [15, 46], at 37 °C for various times. After removal of the ATP solution and PBS washes, cell-containing coverslips were fixed with paraformaldehyde for 10 min, and then with DAPI mount. 12 to 24 hours after the mounting, fixed cells were examined and photographed with fluorescence microscopy (Olympus). Photographed images were analyzed with Image J (NIH). For each condition, 50 to 100 cell images were analyzed for quantifying their number of fluorescent vesicles. Cells without inhibitor treatment were used as controls and their average number was assigned as a relative value of 100% and other treated cells were normalized by and compared to the controls.

### *In vivo* tumor studies on ATP internalization and protein phosphorylation

Two *in vivo* studies were conducted: one for the ATP internalization after NHF-ATP injection and one for protein analysis after sunitinib injection in the presence or absence of ATP. NHF-ATP injection study was done as previously described [15]. For the *in vivo* protein analysis study, A549 tumors grown on a nude mouse were injected with either DMSO (vehicle), 40 μM sunitinib with or without 2 mM ATP, or 2 mM ATP in DMEM in a volume of 100 μL using 1CC syringes with 27G needles. Six minutes after injection, injected mice were euthanized and the tumors were surgically removed and immediately processed for protein isolation. Proteins were analyzed by Western blots exactly the same way as in the *in vitro* assays.

Animal studies were performed in accordance to policies of NIH and Ohio University IACUC.

### Multiple drug resistance (MDR) activity study

ABC transporters are known to be present and functional in A549 [47] and other cancer cell lines. ABCB1, ABCC1, and ABCG2 are expressed in A549 cells participating in MDR [25, 26] while ABCB1, ABCC1 and ABCC2 are expressed in SK-HEP-1 cells [27, 28]. To determine if and how eATP affects MDR in A549 and SK-HEP-1 cells, a fluorometric MDR assay was performed to determine the activity of ABCB1 and ABCC1 using a kit (ab112142, Abcam, Cambridge, MA, USA) and following the assay protocol accompanying the kit. Briefly, A549 or SK-HEP-1 cells (3.0×10^4^ cells/well) were seeded into 96-well clear-bottom black-wall microplates with 100 μl DMEM/well and incubated for 24 hours. Then, the cells were treated with different concentrations of ATP (0.25mM, 0.75mM, and 1mM), or 20 μM Verapamil (ABCB1 inhibitor) [48], 50 μM MK-571 (ABCC1 inhibitor) [49], at 37°C for 2 hours, with no ATP treatment being used as negative controls. Next, 100 μl MDR dye-loading solution was added to each well and incubated at room temperature for 2 hours without light. Fluorescence intensity of cells was detected using a microplate reader (SpectraMax M5, Molecular Devices, USA) at an excitation wavelength of 490 nm and an emission wavelength of 525 nm. All treatments were performed in 6-replicate, and compared to their negative control. The assay detects intensity of fluorescent dye inside cells. A reduction in the dye intensity indicates the MDR (pump) activity.

Next, we wanted to know if inhibition of MDR activity enhances sunitinib’s anticancer efficacy. A549 cell viability with or without MDR inhibitors in the presence or absence of 1 mM eATP was measured using the MTT assay. A549 cells (1×10^4^ cells/well) were seeded in 96-well plates overnight. Then, the cells were treated with either 20 μM sunitinib, 20 μM Verapamil, 50 μM MK-571, or 20 μM sunitinib with each inhibitor of the concentrations mentioned above. All conditions were performed in replicates of 6.

### Purinergic receptor signaling study

Purinergic receptor (PR) signaling requires ATP and is involved in cancer cell metabolism [29, 30]. To determine the role of PR signaling in intracellular ATP level elevation, cell survival, and protein phosphorylation, A549 cells in DMEM supplemented with 1 mM eATP were treated with 50 μM and 100 μM suramin, a PR inhibitor targeting P2 receptors [15, 29] or 1 μM and 2 μM BApTA, a Ca^2+^ chelator that blocks general PR signaling [15, 30]. The treated cells were kept in a 37°C incubator under 5% CO_2_ for various times. After incubation, cells were either subjected to a cell viability (MTT) assay or lysed to analyze their protein phosphorylation after protein isolation. The rationale of this study was to examine the overall PR signaling as a whole, not any specific PR signaling, in the drug resistance.

### siRNA knockdown study

A549 cells were transiently transfected with a verified siRNA specifically against mRNA of p21-activated kinase 1 (PAK1) (from Qiagen, the siRNA sequence can be found in Supplementary Table 2), an important protein involved in macropinocytosis [15, 24]. The transfection was done following the company’s instruction. Scrambled siRNA served as a mock control. Twenty-four hours after the transfection, cells were treated with ATP for 4 hours and then lysed for their intracellular ATP levels measurement. Cell samples treated for 24 hours were used for the protein level measurement of PAK1 by Western blots or MTT assay for their viability / proliferation.

### Cell apoptosis analysis

Apoptotic cell staining was performed using Apoptotic Cells Detection Kit (PromoKine, Heidelberg, Germany) according to the manufacturer’s instructions. Briefly, A549 cells grown on cover slips were treated with or without sunitinib in the presence or absence of eATP for 24 hours. After treatment, cells were washed and stained with FITC-Annexin V solution, which identifies apoptotic cells. Staining solution was then removed, and cells were mounted with Prolong Gold antibody reagent with DAPI (Life technologies), showing cell nuclei in blue. Cells were then photographed with fluorescence microscopy (ECLIPSE E600, Nikon), and images were analyzed with Image J (NIH).

Cleavage of PARP protein is an indicator of apoptosis-induction. PARP cleavage in the sunitinib-treated A549 cells in the presence or absence of eATP was analyzed by Western blots for evidence that eATP affects sunitinib induced apoptosis.

### Metabolic rate measurements

Metabolic analyses were done as previously described [15]. Briefly, glycolysis-related extracellular acidification rate (ECAR) or mitochondrial OXPHOS-related oxygen consumption rate (OCR) of 30,000 A549 cells treated with or without ATP at different concentrations were measured continuously with an XFe 24 Extracellular Flux Analyzer (Seahorse Bioscience). ATP was dissolved in the assay medium and adjusted for pH with NaOH. A549 cells without ATP served as no treatment control.

### Statistical analysis

For all the cell and molecular studies, each experimental condition was performed in triplicates or hexads, and the experiment was repeated at least once. Data is reported as mean ± standard deviation and analyzed using Student’s *t*-test or one-way ANOVA whichever is appropriate. P < 0.05 was considered significant.

## SUPPLEMENTARY MATERIALS FIGURE AND TABLES



## Abbreviations

eATP: extracellular ATP
TKIs: tyrosine kinase inhibitors
iATP: intracellular ATP
MDR: multi-drug resistance
ABC: ATP-binding cassette
NSCLC: non-small cell lung cancer
PDGFR: platelet-derived growth factor receptor
OXPHOS: oxidative phosphorylation
RTKs: receptor tyrosine kinases
NHF-ATP: nonhydrolyzable fluorescent ATP
HMWFD: high molecular weight fluorescent dextrans
RLUs: relative luminescence units
PR: purinergic receptor
PAK1: p21-activated kinase 1
ECAR: extracellular acidification rate
OCR: oxygen consumption rate

## References

[1] Crystal AS, Shaw AT, Sequist LV, Friboulet L, Niederst MJ, Lockerman EL, Frias RL, Gainor JF, Amzallag A, Greninger P, Lee D, Kalsy A, Gomez-Caraballo M (2014). Patient-derived models of acquired resistance can identify effective drug combinations for cancer. Science.

[2] Komarova NL, Wodarz D (2005). Drug resistance in cancer: principles of emergence and prevention. Proc Natl Acad Sci U S A.

[3] Kovalev AA, Tsvetaeva DA, Grudinskaja TV (2013). Role of ABC-cassette transporters (MDR1, MRP1, BCRP) in the development of primary and acquired multiple drug resistance in patients with early and metastatic breast cancer. Exp Oncol.

[4] Marquez B, Van Bambeke F (2011). ABC multidrug transporters: target for modulation of drug pharmacokinetics and drug-drug interactions. Curr Drug Targets.

[5] Vasiliou V, Vasiliou K, Nebert DW (2009). Human ATP-binding cassette (ABC) transporter family. Hum Genomics.

[6] Wilkens S (2015). Structure and mechanism of ABC transporters. F1000Prime Rep.

[7] Pecqueur C, Oliver L, Oizel K, Lalier L, Vallette FM (2013). Targeting metabolism to induce cell death in cancer cells and cancer stem cells. Int J Cell Biol.

[8] Qian Y, Wang X, Liu Y, Li Y, Colvin RA, Tong L, Wu S, Chen X (2014). Extracellular ATP is internalized by macropinocytosis and induces intracellular ATP increase and drug resistance in cancer cells. Cancer Lett.

[9] Zhou Y, Tozzi F, Chen J, Fan F, Xia L, Wang J, Gao G, Zhang A, Xia X, Brasher H, Widger W, Ellis LM, Weihua Z (2012). Intracellular ATP levels are a pivotal determinant of chemoresistance in colon cancer cells. Cancer Res.

[10] Schneider V, Krieger ML, Bendas G, Jaehde U, Kalayda GV (2013). Contribution of intracellular ATP to cisplatin resistance of tumor cells. J Biol Inorg Chem.

[11] Pellegatti P, Raffaghello L, Bianchi G, Piccardi F, Pistoia V, Di Virgilio F (2008). Increased level of extracellular ATP at tumor sites: in vivo imaging with plasma membrane luciferase. PLoS One.

[12] Falzoni S, Donvito G, Di Virgilio F (2013). Detecting adenosine triphosphate in the pericellular space. Interface Focus.

[13] Michaud M, Martins I, Sukkurwala AQ, Adjemian S, Ma Y, Pellegatti P, Shen S, Kepp O, Scoazec M, Mignot G, Rello-Varona S, Tailler M, Menger L (2011). Autophagy-dependent anticancer immune responses induced by chemotherapeutic agents in mice. Science.

[14] Wilhelm K, Ganesan J, Müller T, Dürr C, Grimm M, Beilhack A, Krempl CD, Sorichter S, Gerlach UV, Jüttner E, Zerweck A, Gärtner F, Pellegatti P (2010). Graft-versus-host disease is enhanced by extracellular ATP activating P2×7R. Nat Med.

[15] Qian Y, Wang X, Li Y, Cao Y, Chen X (2016). Extracellular ATP a new player in cancer metabolism: NSCLC cells internalize ATP in vitro and in vivo using multiple endocytic mechanisms. Mol Cancer Res.

[16] Pavlova NN, Thompson CB (2016). The emerging hallmarks of cancer metabolism. Cell Metab.

[17] Chen X, Qian Y, Wu S (2015). The Warburg effect: evolving interpretations of an established concept. Free Radic Biol Med.

[18] Roskoski R (2007). Sunitinib: A VEGF and PDGF receptor protein kinase and angiogenesis inhibitor. Biochem Biophys Res Commun.

[19] Mendel DB, Laird AD, Xin X, Louie SG, Christensen JG, Li G, Schreck RE, Abrams TJ, Ngai TJ, Lee LB, Murray LJ, Carver J, Chan E (2003). In vivo antitumor activity of SU11248, a novel tyrosine kinase inhibitor targeting vascular endothelial growth factor and platelet-derived growth factor receptors: determination of a pharmacokinetic/pharmacodynamic relationship. Clin Cancer Res.

[20] Davidson SM, Papagiannakopoulos T, Olenchock BA, Heyman JE, Keibler MA, Luengo A, Bauer MR, Jha AK, O’Brien JP, Pierce KA, Gui DY, Sullivan LB, Wasylenko TM (2016). Environment impacts the metabolic dependencies of Ras-driven non-small cell lung cancer. Cell Metab.

[21] Liu Y, Cao Y, Zhang W, Bergmeier S, Qian Y, Akbar H, Colvin R, Ding J, Tong L, Wu S, Hines J, Chen X (2012). A small-molecule inhibitor of glucose transporter 1 downregulates glycolysis, induces cell-cycle arrest, and inhibits cancer cell growth in vitro and in vivo. Mol Cancer Ther.

[22] Zhang P, Gao WY, Turner S, Ducatman BS (2003). Gleevec (STI-571) inhibits lung cancer cell growth (A549) and potentiates the cisplatin effect in vitro. Mol Cancer.

[23] Commisso C, Davidson SM, Soydaner-Azeloglu RG, Parker SJ, Kamphorst JJ, Hackett S, Grabocka E, Nofal M, Drebin JA, Thompson CB, Rabinowitz JD, Vander Heiden MG (2013). Macropinocytosis of protein is an amino acid supply route in Ras-transformed cells. Nature.

[24] Redelman-Sidi G, Iyer G, Solit DB, Glickman MS (2013). Oncogenic activation of Pak1-dependent pathway of macropinocytosis determines BCG entry into bladder cancer cells. Cancer Res.

[25] Chen Y, Scully M, Petralia G, Kakkar A (2014). Binding and inhibition of drug transport proteins by heparin: a potential drug transporter modulator capable of reducing multidrug resistance in human cancer cells. Cancer Biol Ther.

[26] Szakács G, Annereau JP, Lababidi S, Shankavaram U, Arciello A, Bussey KJ, Reinhold W, Guo Y, Kruh GD, Reimers M, Weinstein JN, Gottesman MM (2004). Predicting drug sensitivity and resistance: profiling ABC transporter genes in cancer cells. Cancer Cell.

[27] Yang T, Zheng Z, Li X, Li Z, Wang Y, Geng Y, Bai L, Zhang X (2013). MiR-223 modulates multidrug resistance via downregulation of ABCB1 in hepatocellular carcinoma cells. Exp Biol Med.

[28] Zhou Y, Ling XL, Li SW, Li XQ, Yan B (2010). Establishment of a human hepatoma multidrug resistant cell line in vitro. World J Gastroenterol.

[29] Cheng SE, Lee IT, Lin CC, Wu WL, Hsiao LD, Yang CM (2013). ATP mediates NADPH oxidase/ROS generation and COX-2/PGE2 expression in A549 cells: role of P2 receptor-dependent STAT3 activation. PLoS One.

[30] Burnstock G, Di Virgilio F (2013). Purinergic signalling and cancer. Purinergic Signal.

[31] Matsumoto S, Yasui H, Mitchell JB, Krishna MC (2010). Imaging cycling tumor hypoxia. Cancer Res.

[32] Cárdenas-Navia LI, Mace D, Richardson RA, Wilson DF, Shan S, Dewhirst MW (2008). The pervasive presence of fluctuating oxygenation in tumors. Cancer Res.

[33] Toffoli S, Michiels C (2008). Intermittent hypoxia is a key regulator of cancer cell and endothelial cell interplay in tumours. FEBS J.

[34] Koppenol WH, Bounds PL, Dang CV (2011). Otto Warburg’s contributions to current concepts of cancer metabolism. Nat Rev Cancer.

[35] Pfeiffer T, Schuster S, Bonhoeffer S (2001). Cooperation and competition in the evolution of ATP-producing pathways. Science.

[36] Sounni NE, Noel A (2013). Targeting the tumor microenvironment for cancer therapy. Clin Chem.

[37] Balkwill FR, Capasso M, Hagemann T (2012). The tumor microenvironment at a glance. J Cell Sci.

[38] Tape CJ, Ling S, Dimitriadi M, McMahon KM, Worboys JD, Leong HS, Norrie IC, Miller CJ, Poulogiannis G, Lauffenburger DA, Jørgensen C (2016). Oncogenic KRAS regulates tumor cell signaling via stromal reciprocation. Cell.

[39] Stehle G, Sinn H, Wunder A, Schrenk HH, Stewart JC, Hartung G, Maier-Borst W, Heene DL (1997). Plasma protein (albumin) catabolism by the tumor itself–implications for tumor metabolism and the genesis of cachexia. Crit Rev Oncol Hematol.

[40] Galluzzi L, Buqué A, Kepp O, Zitvogel L, Kroemer G (2017). Immunogenic cell death in cancer and infectious disease. Nat Rev Immunol.

[41] Kamphorst JJ, Nofal M, Commisso C, Hackett SR, Lu W, Grabocka E, Vander Heiden MG, Miller G, Drebin JA, Bar-Sagi D, Thompson CB, Rabinowitz JD (2015). Human pancreatic cancer tumors are nutrient poor and tumor cells actively scavenge extracellular protein. Cancer Res.

[42] Patel A, Malinovska L, Saha S, Wang J, Alberti S, Krishnan Y, Hyman AA (2017). ATP as a biological hydrotrope. Science.

[43] Fabian MA, Biggs WH, Treiber DK, Atteridge CE, Azimioara MD, Benedetti MG, Carter TA, Ciceri P, Edeen PT, Floyd M, Ford JM, Galvin M, Gerlach JL (2005). A small molecule–kinase interaction map for clinical kinase inhibitors. Nat Biotechnol.

[44] Becher I, Savitski MM, Savitski MF, Hopf C, Bantscheff M, Drewes G (2013). Affinity profiling of the cellular kinome for the nucleotide cofactors ATP, ADP, and GTP. ACS Chem Biol.

[45] Morrone FB, Oliveira DL, Gamermann P, Stella J, Wofchuk S, Wink MR, Meurer L, Edelweiss MI, Lenz G, Maria A, Battastini O (2006). *In vivo* glioblastoma growth is reduced by apyrase activity in a rat glioma model. BMC Cancer.

[46] Ivanov AI (2008). Pharmacological inhibition of endocytic pathways: is it specific enough to be useful?. Methods Mol Biol.

[47] Sakamoto A, Matsumaru T, Yamamura N, Suzuki S, Uchida Y, Tachikawa M, Terasaki T (2015). Drug transporter protein quantification of immortalized human lung cell lines derived from tracheobronchial epithelial cells (Calu-3 and BEAS2-B), bronchiolar–alveolar cells (NCI-H292 and NCI-H441), and alveolar type II-like cells (A549) by liquid chromatography–tandem mass spectrometry. J Pharm Sci.

[48] Scharenberg CW, Harkey MA, Torok-Storb B (2002). The ABCG2 transporter is an efficient Hoechst 33342 efflux pump and is preferentially expressed by immature human hematopoietic progenitors. Blood.

[49] Lespine A, Dupuy J, Orlowski S, Nagy T, Glavinas H, Krajcsi P, Alvinerie M (2006). Interaction of ivermectin with multidrug resistance proteins (MRP1, 2 and 3). Chem Biol Interact.

