# Supplementary material for: Extracellular ATP, as an energy and phosphorylating molecule, induces different types of drug resistances in cancer cells through ATP internalization and intracellular ATP level increase
Source: Oncotarget. 2017 Sep 23;8(50):87860–77. doi: 10.18632/oncotarget.21231 (PMC5675678; doi:10.18632/oncotarget.21231)
Supplement: Supplementary file 1 [file oncotarget-08-87860-s001.pdf]

Extracellular ATP, as an energy and phosphorylating molecule, induces different types of drug resistances in cancer cells through ATP internalization and intracellular ATP level increase

SUPPLEMENTARY MATERIALS

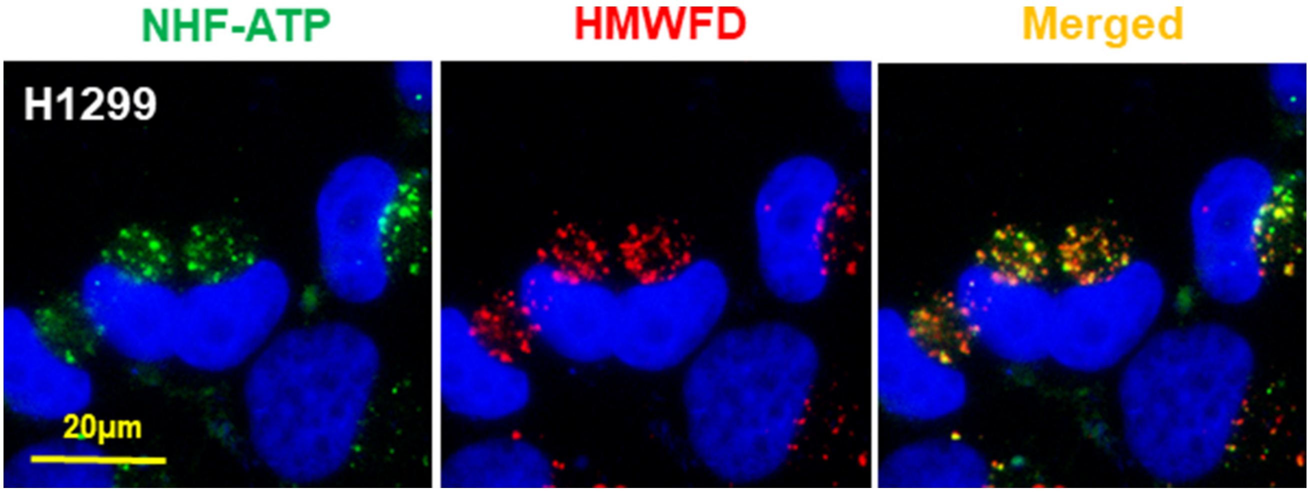

Supplementary Figure 1: H1299 cells internalize NHF-ATP and HMWFD by macropinocytosis. Human non-small cell lung cancer H1299 cells grown on coverslips were incubated with NHF-ATP (green) and HMWFD (red) as described in Figure 2 for A549 cells. After incubation, and fixation, cells were visualized with fluorescent microscopy and analyzed by Image J. Yellowish spots in the merged photo indicate the co-internalization of NHF-ATP and HMWFD and intracellular location of macropinosomes.

Supplementary Table 1: Concentrations of anticancer drugs used for each cell line (µM)

	Sunitinib	Sorafenib	Gefitinib	Erlotinib	Imatinib	Paclitaxel	Cisplatin	Doxorubicin
A549 (lung)	20	20	20	40	10	2	120	5
MCF7 (breast)	20	20	20	40	30	2	30	5
HT-29 (colon)	20	20	20	40	10	2	120	1
PANC-1 (pancreas)	20	20	30	40	30	2	30	5
SK-HEP-1 (liver)	20	20	40	60	50	2	60	5

Supplementary Table 2: iATP elevation induced by eATP and ABC transporters expressed by five cancer cell lines

	iATP elevation	ABC transporters expression
A549 (lung)	Yes	ABCB1, ABCC1, ABCG2 [1, 2]
SK-HEP-1 (liver)	Yes	ABCB1, ABCC1, ABCC2 [3, 4]
MCF7 (breast)	Yes	ABCG2 [5]
HT-29 (colon)	Not detectable	ABCC1, ABCC3 [6, 7]
PANC-1 (pancreas)	Yes	ABCC1, ABCG2 [8, 9]

Note: References are listed below.

1. Scharenberg CW, Harkey MA, Torok-Storb B. The ABCG2 transporter is an efficient Hoechst 33342 efflux pump and is preferentially expressed by immature human hematopoietic progenitors. Blood. 2002; 99: 507–12.
2. Chen Y, Scully M, Petralia G, Kakkar A. Binding and inhibition of drug transport proteins by heparin: a potential drug transporter modulator capable of reducing multidrug resistance in human cancer cells. Cancer Biol Ther. 2014; 15: 135–45. https://doi.org/10.4161/cbt.27148.
3. Yang T, Zheng Z, Li X, Li Z, Wang Y, Geng Y, Bai L, Zhang X. MiR-223 modulates multidrug resistance via downregulation of ABCB1 in hepatocellular carcinoma cells. Exp Biol Med. 2013; 238: 1024–32. https://doi.org/10.1177/1535370213497321.
4. Zhou Y, Ling XL, Li SW, Li XQ, Yan B. Establishment of a human hepatoma multidrug resistant cell line *in vitro*. World J Gastroenterol. 2010; 16: 2291–7.
5. Horsey AJ, Cox MH, Sarwat S, Kerr ID. The multidrug transporter ABCG2: still more questions than answers multidrug transporters in biology. Biochem Soc Trans. 2016; 44, 824–30. https://doi.org/10.1042/BST20160014.
6. Cummings J, Zelcer N, Allen JD, Yao D, Boyd G, Maliepaard M, Friedberg TH, Smyth JF, Jodrell DI. Glucuronidation as a mechanism of intrinsic drug resistance in colon cancer cells: contribution of drug transport proteins. Biochem Pharmacol. 2004; 67: 31–9.
7. Kobayashi M, Funayama R, Ohnuma S, Unno M, Nakayama K. Wnt-β-catenin signaling regulates ABCC3 (MRP3) transporter expression in colorectal cancer. Cancer Sci. 2016; 107: 1776–84. https://doi.org/10.1111/cas.13097.
8. Pan YZ, Zhou A, Hu Z, Yu AM. Small nucleolar RNA-derived microRNA hsa-miR-1291 modulates cellular drug disposition through direct targeting of ABC transporter ABCC1. Drug Metab Dispos. 2013; 41: 1744–51. https://doi.org/10.1124/dmd.113.052092.
9. Wang F, Xue X, Wei J, An Y, Yao J, Cai H, Wu J, Dai C, Qian Z, Xu Z, Miao Y. hsa-miR-520h downregulates ABCG2 in pancreatic cancer cells to inhibit migration, invasion, and side populations. Br J Cancer. 2010; 103: 567–74. https://doi.org/10.1038/sj.bjc.6605724.

Supplementary Table 3: ABC transporters responsible for the efflux of different drugs

	Sunitinib	Sorafenib	Gefitinib	Erlotinib	Imatinib	Paclitaxel	Cisplatin	Doxorubicin
ABC transporters	ABCB1<br>ABCC1<br>ABCG2<br>[1–3]	ABCG2 [4]	ABCB1<br>ABCG2<br>[5, 6]	ABCG2<br>[7]	ABCB1<br>ABCG2<br>[8, 9]	ABCB1<br>ABCC1 [10]	ABCC2<br>ABCC3<br>[10]	ABCB1<br>ABCC2<br>ABCC3<br>ABCC5<br>ABCC6 [10]

Note: References are listed below.

1. Amiri-Kordestani L, Basseville A, Kurdziel K, Fojo AT, Bates SE. Targeting MDR in breast and lung cancer: discriminating its potential importance from the failure of drug resistance reversal studies. Drug Resist Updat. 2012; 15: 50–61. https://doi.org/10.1016/j.drup.2012.02.002.
2. Tang SC, Lagas JS, Lankheet NA, Poller B, Hillebrand MJ, Rosing H, Beijnen JH, Schinkel AH. Brain accumulation of sunitinib is restricted by P-glycoprotein (ABCB1) and breast cancer resistance protein (ABCG2) and can be enhanced by oral elacridar and sunitinib coadministration. Int J Cancer. 2012; 130: 223–33. https://doi.org/10.1002/ijc.26000.
3. Noguchi K, Katayama K, Sugimoto Y. Human ABC transporter ABCG2/BCRP expression in chemoresistance: basic and clinical perspectives for molecular cancer therapeutics. Pharmgenomics Pers Med. 2014; 7: 53. https://doi.org/10.2147/PGPM.S38295.
4. Lagas JS, van Waterschoot RA, Sparidans RW, Wagenaar E, Beijnen JH, Schinkel AH. Breast cancer resistance protein and P-glycoprotein limit sorafenib brain accumulation. Mol Cancer Ther. 2010; 9: 319–26. https://doi.org/10.1158/1535-7163.MCT-09-0663.
5. Agarwal S, Sane R, Gallardo JL, Ohlfest JR, Elmquist WF. Distribution of gefitinib to the brain is limited by P-glycoprotein (ABCB1) and breast cancer resistance protein (ABCG2)-mediated active efflux. J Pharmacol Exp Ther. 2010 ; 334: 147–55. https://doi.org/10.1124/jpet.110.167601.
6. Elkind NB, Szentpétery Z, Apáti Á, Özvegy-Laczka C, Várady G, Ujhelly O, Szabó K, Homolya L, Váradi A, Buday L, Kéri G, Német K, Sarkadi B. Multidrug transporter ABCG2 prevents tumor cell death induced by the epidermal growth factor receptor inhibitor iressa (ZD1839, Gefitinib). Cancer Res. 2005; 65: 1770–7. https://doi.org/10.1158/0008-5472.CAN-04-3303.
7. Natarajan K, Xie Y, Baer MR, Ross DD. Role of breast cancer resistance protein (BCRP/ABCG2) in cancer drug resistance. Biochem Pharmacol. 2012; 83: 1084–103. https://doi.org/10.1016/j.bcp.2012.01.002.
8. Sun YL, Patel A, Kumar P, Chen ZS. Role of ABC transporters in cancer chemotherapy. Chin J Cancer. 2012; 31: 51–7. https://doi.org/10.5732/cjc.011.10466.
9. Stacy AE, Jansson PJ, Richardson DR. Molecular pharmacology of ABCG2 and its role in chemoresistance. Mol Pharmacol. 2013; 84: 655–69. https://doi.org/10.1124/mol.113.088609.
10. Choi CH. ABC transporters as multidrug resistance mechanisms and the development of chemosensitizers for their reversal. Cancer Cell Int. 2005; 5: 30. https://doi.org/10.1186/1475-2867-5-30.
